# Intraoperative radiotherapy combined with spinal stabilization surgery—a novel treatment strategy for spinal metastases based on a first single-center experiences

**DOI:** 10.1007/s11060-024-04688-1

**Published:** 2024-04-23

**Authors:** P. Krauss, C. L. Wolfert, B. Sommer, B. Stemmer, G. Stueben, K. H. Kahl, E. Shiban

**Affiliations:** 1 Department of Neurosurgery, University Hospital Augsburg, Stenglinstrasse 2, 86156 Augsburg, Germany; 2 Department of Radio Oncology, University Hospital Augsburg, Stenglinstrasse 2, 86156 Augsburg, Germany

**Keywords:** Intraoperative radiotherapy, Spinal metastasis, IORT, Spinal stabilization surgery

## Abstract

**Introduction:**

Current treatment of spinal metastases (SM) aims on preserving spinal stability, neurological status, and functional status as well as achieving local control. It consists of spinal surgery followed by radiotherapy and/or systemic treatment. Adjuvant therapy usually starts with a delay of a few weeks to prevent wound healing issues. Intraoperative radiotherapy (IORT) has previously been successfully applied during brain tumor, breast and colorectal carcinoma surgery but not in SM, including unstable one, to date. In our case series, we describe the feasibility, morbidity and mortality of a novel treatment protocol for SM combining stabilization surgery with IORT.

**Methods:**

Single center case series on patients with SM. Single session stabilization by navigated open or percutaneous procedure using a carbon screw-rod system followed by concurrent 50 kV photon-IORT (ZEISS Intrabeam). The IORT probe is placed via a guide canula using navigation, positioning is controlled by IOCT or 3D-fluroscopy enabling RT isodose planning in the OR.

**Results:**

15 (8 female) patients (71 ± 10y) received this treatment between 07/22 and 09/23. Median Spinal Neoplastic Instability Score was 8 [7–10] IQR. Most metastasis were located in the thoracic (*n* = 11, 73.3%) and the rest in the lumbar (*n* = 4, 26.7%) spine. 9 (60%) patients received open, 5 (33%) percutaneous stabilization and 1 (7%) decompression only. Mean length of surgery was 157 ± 45 min. Eleven patients had 8 and 3 had 4 screws placed. In 2 patients radiotherapy was not completed due to bending of the guide canula with consecutive abortion of IORT. All other patients received 8 Gy isodoses at mdn. 1.5 cm [1.1–1.9, IQR] depth during 2-6 min. The patients had Epidural Spinal Cord Compression score 1a-3. Seven patients (46.7%) experienced adverse events including 2 surgical site infection (one 65 days after surgery).

**Conclusion:**

50 kV photon IORT for SM and consecutive unstable spine needing surgical intervention is safe and feasible and can be a promising technique in selected cases.

## Introduction

The incidence of spinal metastases (SM) is reported to be around 16% in solid tumors and result in meaningful impairment of quality of life (QOL) due to pain or neurological deficits [1–4]. The current treatment of SM involves spinal surgery and adjuvant radiotherapy (RT) and systemic therapy (CTX) [5]. Surgery aims to protect the spinal cord (SC) via decompression of epidural tumor masses and separation surgery to enable safe RT respecting the SC as an “organ at risk” [1, 6]. Furthermore, spinal stabilization surgery is essential to prevent progressive spinal deformity and improve pain from pathological fractures [7]. RT is crucial for local tumor control. However, if surgery is performed, RT and CTX are usually delayed several weeks to prevent wound healing issues and enable recovery of the patient but fast transition to adjuvant therapy is known to improve clinical outcome [8–10]. Intraoperative radiotherapy (IORT) has become an emerging treatment option in breast and colorectal cancer as well as brain metastases (BM) [11–14]. IORT for SM has not yet been established as a routine treatment. Recent technological advances enabled the successful combination of kyphoplasty with IORT for patients with stable SM presenting with pain [9, 15, 16]. In patients with unstable SM or severe deformity, kyphoplasty is not an option as does spinal stabilization surgery with screw-rod instrumentation (with or without vertebral body replacement) is warranted to prevent progression of spinal deformity [17].

In our case series we present a novel treatment protocol for spinal stabilization surgery combined with IORT in SM as single session procedure. We describe the technical details and report preliminary morbidity and mortality results.

## Methods

### Ethics approval

The study protocol was approved by the local ethics committee (UKA/LMU; N°:23–0622) in accordance to the Declaration of Helsinki. For this retrospective observational study, no individual informed consent was necessary according to the ethics committee’s guidelines and regulations.

### Study design

We performed a retrospective analysis of prospectively collected patient-specific clinical records in a single tertiary neurosurgical center. The analyzed parameters included age, sex, the Spinal Instability Neoplastic Score (SINS), the Epidural Spinal Cord Compression Scale (ESCC), length of surgery (LOS) and adverse events (AEs) up to the 30th postoperative day (POD) according to the Clavien-Dindo Grading system (CDG) [18–20].

### Patient selection

Patients were treated based on an individual treatment protocol and had to specifically consent to IORT as its not a standard of care yet. Inclusion criteria were SINS ≥ 7 and a signed informed consent. Indication for stabilization and IORT was based on an interdisciplinary neurooncological board and after anesthesiologic evaluation for perioperative morbidity.

### Statistics

Statistical analysis was performed using SPSS Statistics™ (version 25, IBM Corp, Armonk, New York, USA). Data in text and graphs are shown as mean and standard deviation (SD) for continuous data and as median and interquartile range for ordinal data.

### Surgical procedure

**Fig. 1:**
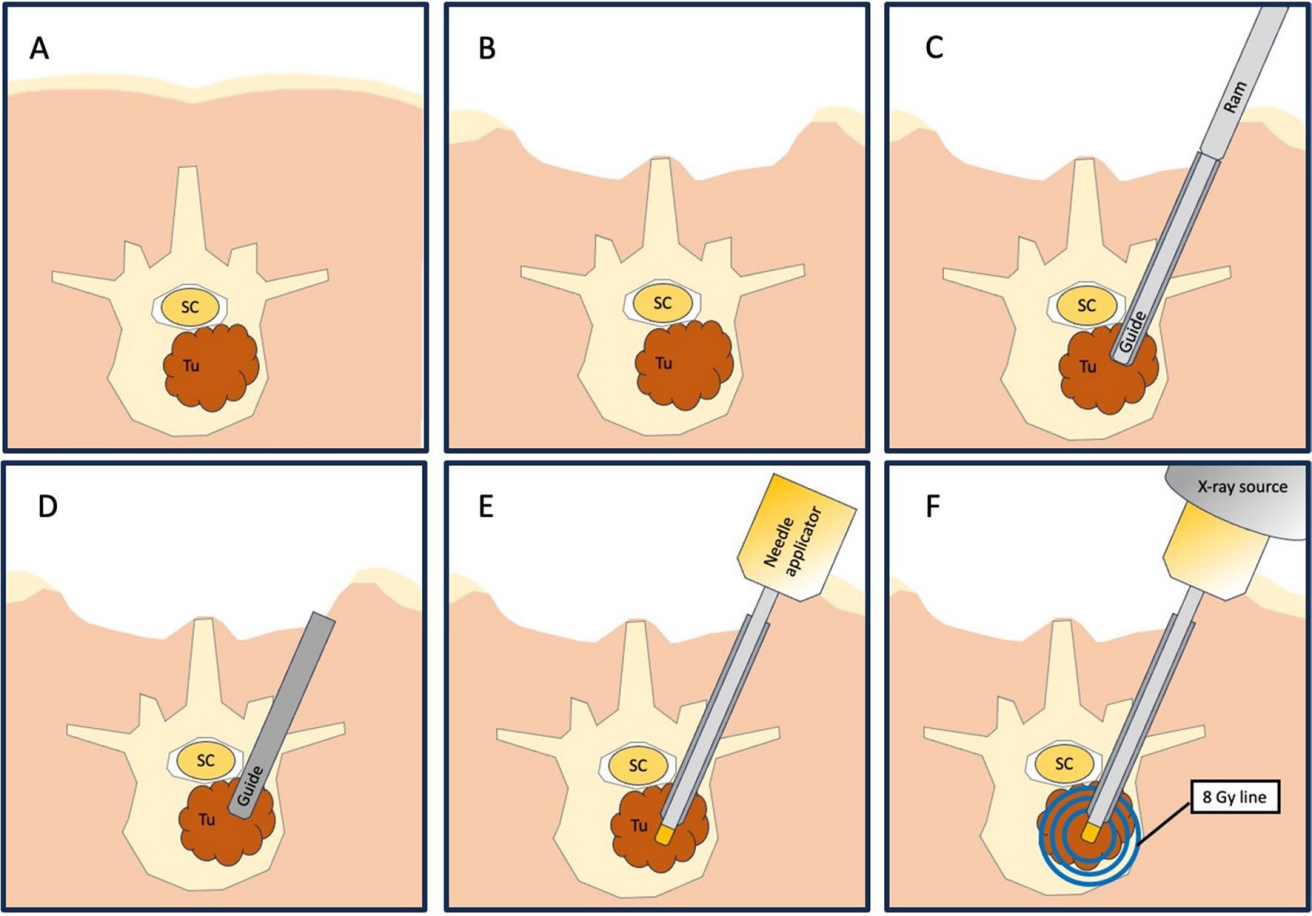
Illustrated concept of intraoperative radiotherapy in spin surgery: **A**) Vertebral tumor mass **B**) Subcutaneous dissection for transmuscular tube placement **C**) Guide tube placement with ram **D**) Ram is removed, tub lies in tumor center **E**) Needle applicator is placed along the guide **F**) Radiation through the needle applicator respecting the “8 Gray line” to the spinal cord. Tu = tumor; SC = spinal cord

**Fig. 2:**
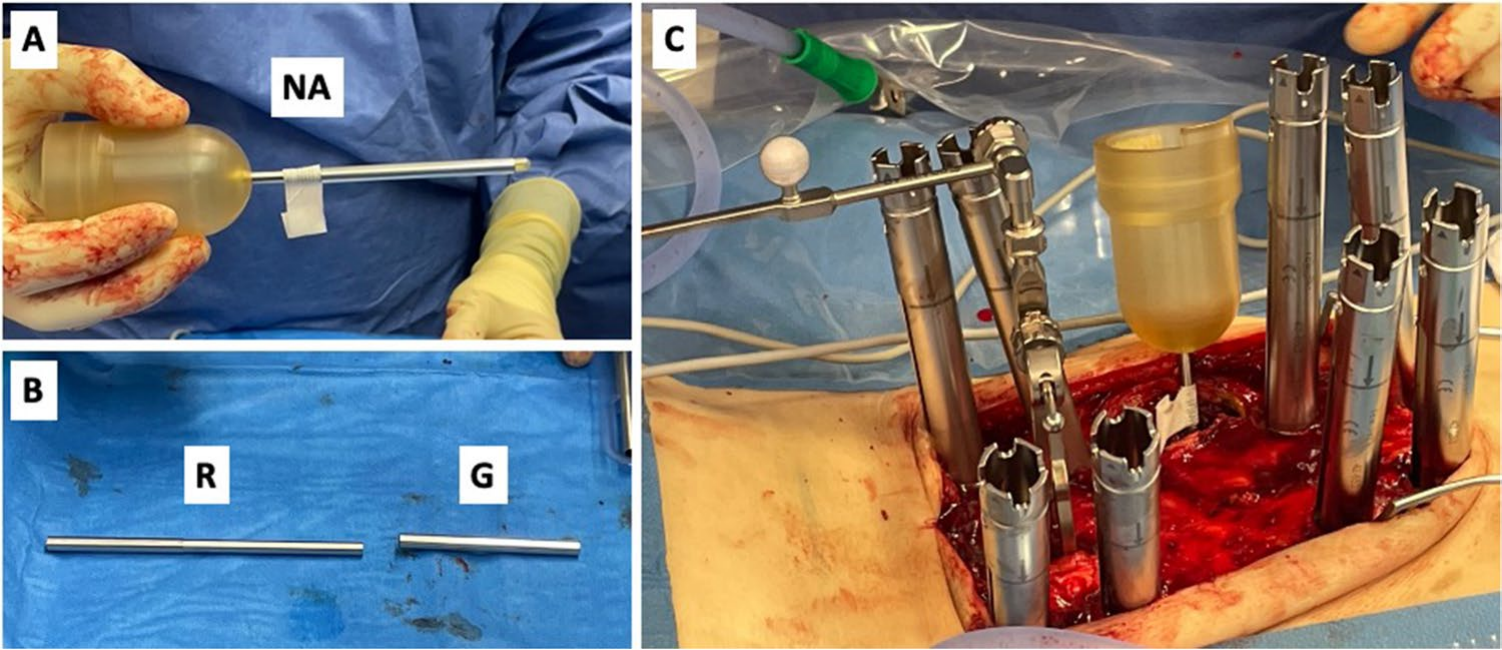
** A**) The insertion depth of the needle applicator (NA) is marked with a sterile strip. **B**) The ram (R) and guide (G) for the needle applicator. **C**) intraoperative situs with percutaneous screw placement setup and needle applicator in place

**Fig. 3:**
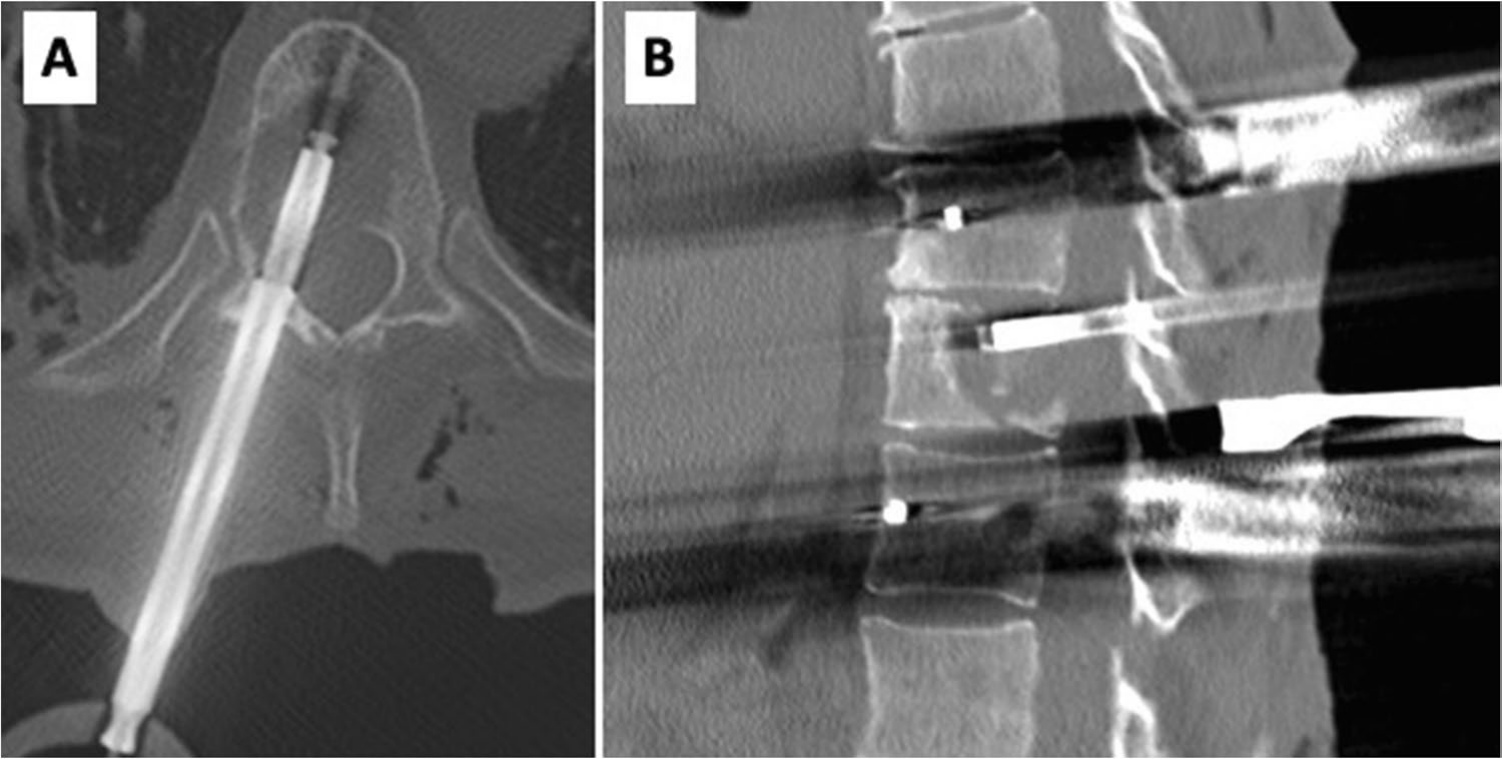
**A**) axial CT scan with intravertebral placement of guide and needle applicator in axial and **B**) sagittalplane

**Fig. 4:**
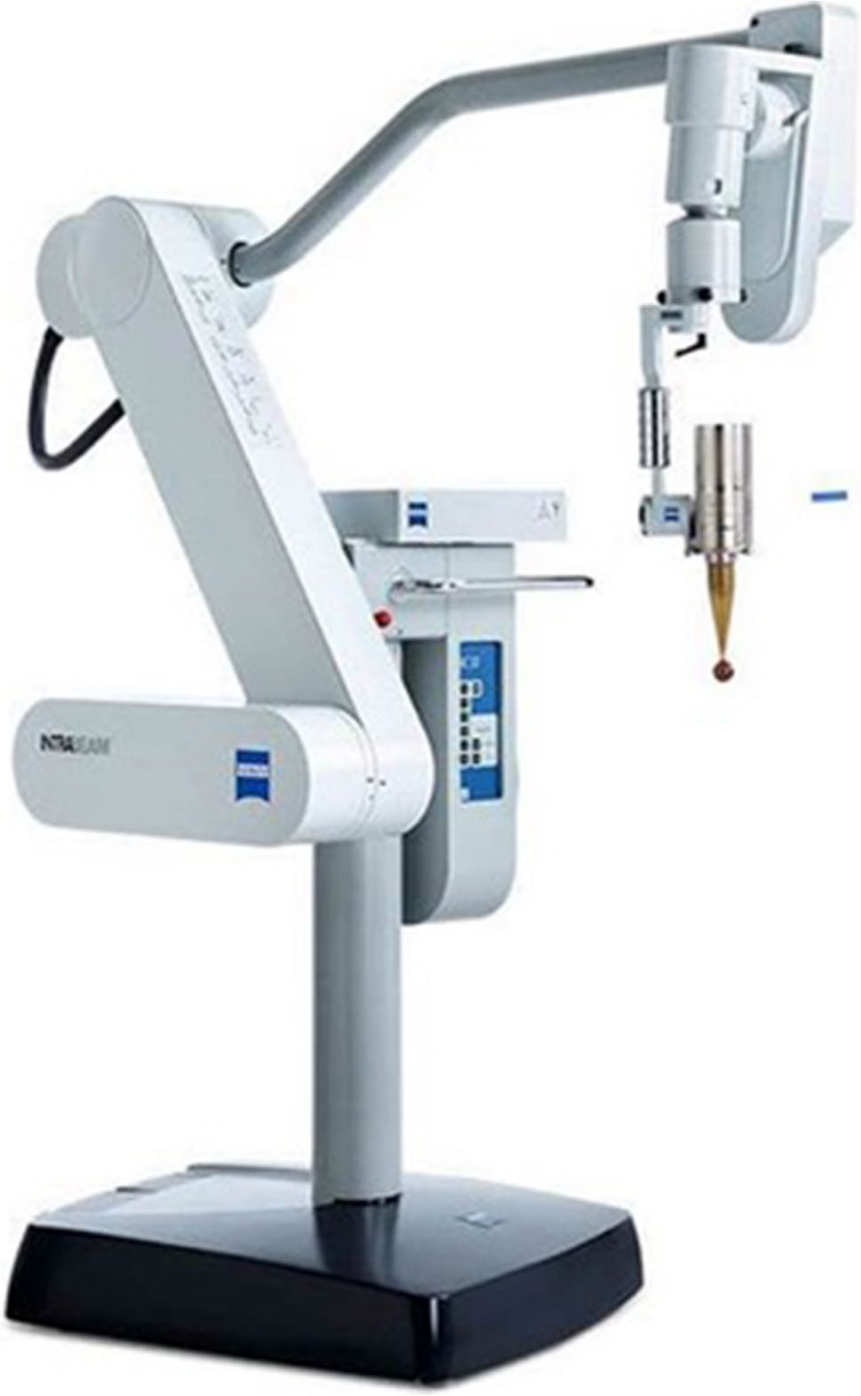
The INTRABEAM® floor stand

**Fig. 5:**
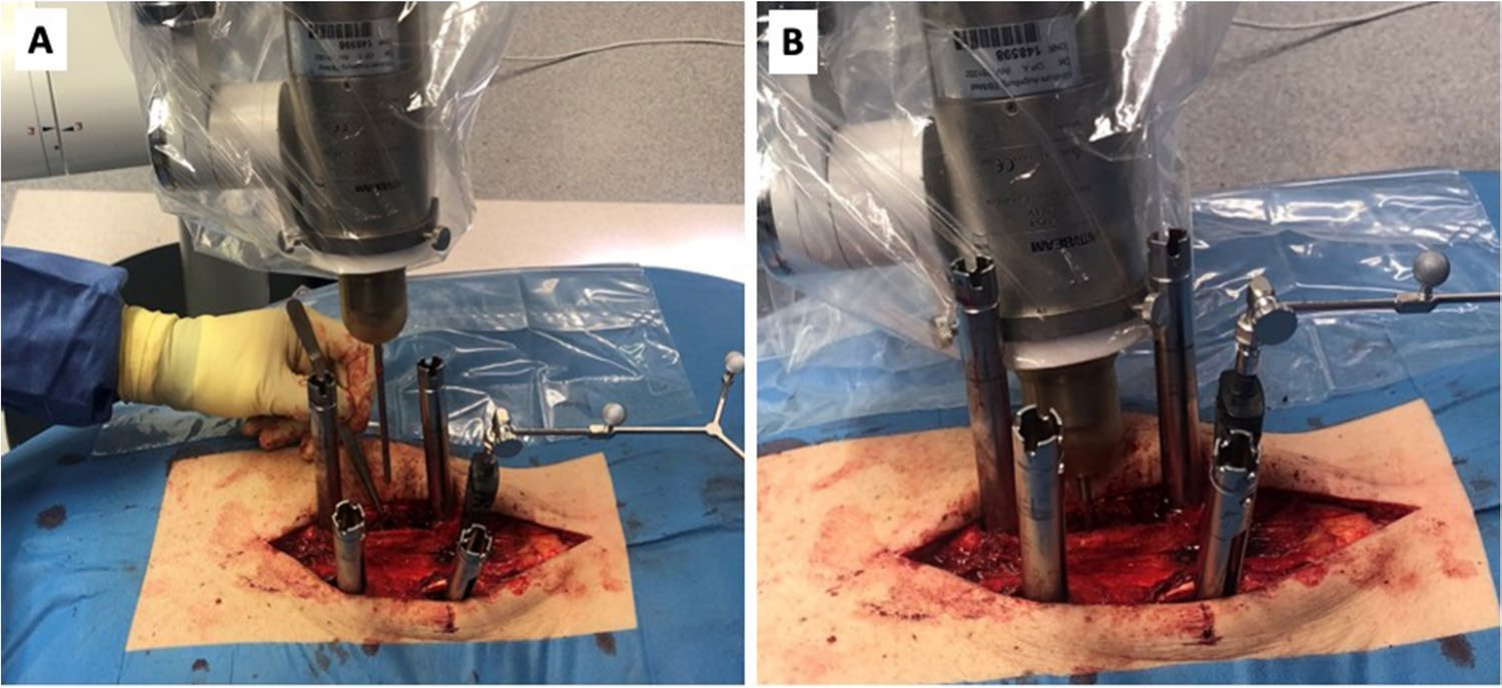
Intraoperative situs with percutaneous screw placement setup and insertion of sterile needle applicator with INTRABEAM® (**A**+**B**)

Surgery was performed in general anesthesia with the patients in prone position. After sterile disinfection and draping a median skin incision was performed for both open and percutaneous pedicle screw placement. The most caudal spinal process was exposed to fixate the array for spinal navigation (BRAINLAB, Germany). After the navigation scan (intraoperative computed tomography (CT) (Healthineers, Germany) or 3D-Flurosocpy (Arcadis Healthineers, Germany)), carbon screw (ICOTEC Vader®, CT, US) placement was performed using a k-wire guided technique. A transpedicular navigated biopsy at the index vertebra was generally performed using a 3.2 mm drill, aiming at the center of the metastasis. The trajectory was enlarged via a 7 mm thread cutter and will serve for the guiding canula (4.4 mm inner-diameter, 55 m length) for the RT needle applicator (INTRABEAM®, Carl Zeiss Meditec, Germany) using its ram (Figs. 1C and 2A). After removal of the ram (Fig. 1D), the needle applicator (4.4 mm outer-diameter, 94 mm length) is inserted until the plastic tip at the distal end of the guiding canula is completely overlooking the guiding cannula (marked with a sterile strip) (Figs. 1E and 2B). After screw and needle applicator placement (Fig. 2C), the correct positions were verified via an additional CT or 3D-fluoroscopy scan (Fig. 3A and B). After verification of the correctly positioned needle applicator tip, the radiation doses via 50 kV-X-rays are calculated to reach 8 Gy isodoses at the border of the tumor tissue, sparing the SC as organ at risk (Fig. 1F) as previously described [21]. The maximum dose applied to the spinal cord is 8 Gy with 50 kV x-rays. Keeping in mind that 50 kV x-rays have a relative biological efficacy (RBE) of 1.3, this is isoeffective with approximately 10 Gy with 6MV photons beam radiotherapy (EBRT). The main difference is the dosing concept. If using conventional external beam radiotherapy (EBRT), you aim to achieve homogeneous dose distribution within your target volume. If you apply EBRT as SBRT, you allow dose inhomogeneity with a maximum of 120–150% of the prescribed dose. The dosing concept of this IORT is a single channel brachytherapy dosimetry. The dose is calculated to a distance from the tip of the needle applicator. You literally irradiate from the center of the metastasis to its periphery. This leads to extremely high doses around the needle tip of 250% to 500% of the prescribed dose. With the extremely high dose in the central part of the metastasis we generate a profound immunological signal to the immune system. For dose calculation we measure the distance from the needle tip to the spinal canal in our intraoperative CT or 3D fluoroscopy scan with the applicator in place. The needle applicator is put on the INTRABEAM® floorstand (Fig. 4) after sterile draping and inserted into the guide canula (Figs. 1F and 5A and B). It is of utmost importance to avoid bending of the needle applicator impeding sufficient X-ray emergence at the tip of the applicator, as this will trigger the automatic abortion of the radiation process. Especially in lumbar spine and more oblique trajectories, soft tissue needs to be separated from the needle applicator to minimize pressure and bending. The anesthesiologist is advised to reduce the patient’s inspiratory volume to avoid excessive movement in the thoracic spine, which can lead to needle bending, too. Then, radiation is started and supervised via a control station outside the theatre. During IORT, theater personnel is allowed to be in the theatre wearing adequate radiation protection. Mean radiation time is 5 min whilst the estimated time to install the INTRABEAM is 10 min. After IORT, the needle applicator and guide canula are removed (single use), the carbon rods inserted and fixated, and the surgical site is closed in the usual manner.

## Results

### Patient population

We were able to include 15 patients, 8 females, treated between 07/22 and 09/23. Mean age was 71 ± 10 years. One patient underwent decompression and IORT alone without stabilization. No patient had previous RT at the index level. See Table 1 for baseline characteristics.

**Table 1:** Patient characteristics. Data is displayed as mean ± standard deviation or median [interquartile range]; Pat = patient, y = years, SINS = spinal instability neoplastic score, ESCC = epidural spinal cord compression scale, Proc = Procedure, Perc = transmuscular, Dec. = decompression, LOS = length of surgery, min. = minutes, NSCLC = non-small cell lung cancer, d = days

Pat	Age (y)	level	SINS	ESCC	Proc	Proc	LOS (min.)	Entity	Follow up (d)
1	68	T 10	10	2	Perc	T9-11	115	Breast cancer	95
2	80	T 12	9	1a	Open	T10-11-L1-2	117	NSCLC	283
3	66	L 3	10	1a	Open	L1-2–4-5	155	Breast cancer	121
4	66	T 5	7	1c	Perc	T3-4–6-7	98	Colorectal cancer	22
5	74	T 4	9	2	Perc	T2-3–5-6	225	Myeloma	109
6	59	T 4	11	3	Open	T3-4–6-7	149	Breast cancer	89
7	79	L 5	7	1a	Open	L3-4-S1-2	248	Colorectal cancer	29
8	83	T 10	7	2	Open	T8-9–11-12	209	Renal cell cancer	23
9	57	T 4	4	3	Dec. Only	T4	93	Colorectal cancer	51
10	73	T 5	7	1c	Open	T3-4–6-7	157	Urothel cancer	20
11	56	T 5	8	2	Open	T4-6	122	NSCLC	LTF
12	82	T 7	7	2	Perc	T5-6–8-9	159	Prostate cancer	50
13	83	L 1	10	3	Perc	T11-12-L2-3	158	NSCLC	67
14	58	L 5	7	3	Open	L4-S1	167	Myeloma	99
15	79	T 12	7	2	Open	T10-11-L1-2	186	Renal cell cancer	20
	71 ± 10		7 [7-10]				157 ± 45		77 ± 69

### Functional status

The median functional status (KPS) was 80% [70–90 IQR] before surgery and could be preserved upon discharge (median KPS 80% [70–90 IQR]); *p* = 0.76) at discharge. Two patient showed worsening of the functional status. One from KPS 50% to 20% and another one from 70 to 60%. In five patients, the functional status improved by 10%. These changes could objectively not be led back to the IORT. Mean length of hospital stay 9 days (± 7 SD). Patients were either discharged home or the department of oncology for systemic therapy.

### Adverse events

In 7/15 patients, a total of 10 adverse events (AE) occurred up until 30 days postop. One patient needed surgical revision for surgical site infection (SSI). Despite surgery, functional status worsened significantly and the patient died on day 22 due to systemic infection. Low -molecular-weight heparin for deep venous thrombosis or antibiotic treatment for pneumonia was necessary in two other patients. One patient experienced progressive tumor growth with increasing paraparesis during this short period of follow-up necessitating recurrent surgical decompression at the index level. This same patient experienced a late SSI at POD 65 needing surgical revision (for further details see Table 2). Patients who developed AEs did not differ significantly from patients who did not experience AEs according to age, LOS, number of implanted screws, SINS or ESCC (for further details see Table 3).

**Table 2:** Adverse events; SSI = surgical site infection, DVT = deep venous thrombosis, CDG= Clavien Dindo grading, d = days

Pat	Adverse Event	CDG	Time after Surgery (d)
3	Seroma	1	16
4	SSI, Death	3b, 5	20, 22
6	DVT	2	18
9	Pneumonia, Port Infection	2, 2	15, 29
12	Seroma	1	11
13	Neurological decline, SSI	3b, 3b	15, **65**
15	Worsening of General Status, Death	4, 5	21

**Table 3:** Characteristics in patients experiencing adverse events. Data is displayed as mean ± standard deviation or median [interquartile range] Y = years, LOS = length of surgery, min = minutes, n = number, SINS = spinal neoplastic instability score, pt = points, ESCC = epidural spinal cord compression scale, AE = adverse event, ns = non-significant

	No AE	AE	p-value
Age (y)	71 ± 10	70 ± 11	ns
LOS (min)	170 ± 52	143 ± 34	ns
Screws (n)	6.5 ± 2	6.9 ± 3	ns
SINS (pt)	7.5 [7-9]	7 [7-10]	ns
ESCC	2 [1b-2]	2 [1b-3]	ns

### Illustrative case

**Fig. 6:**
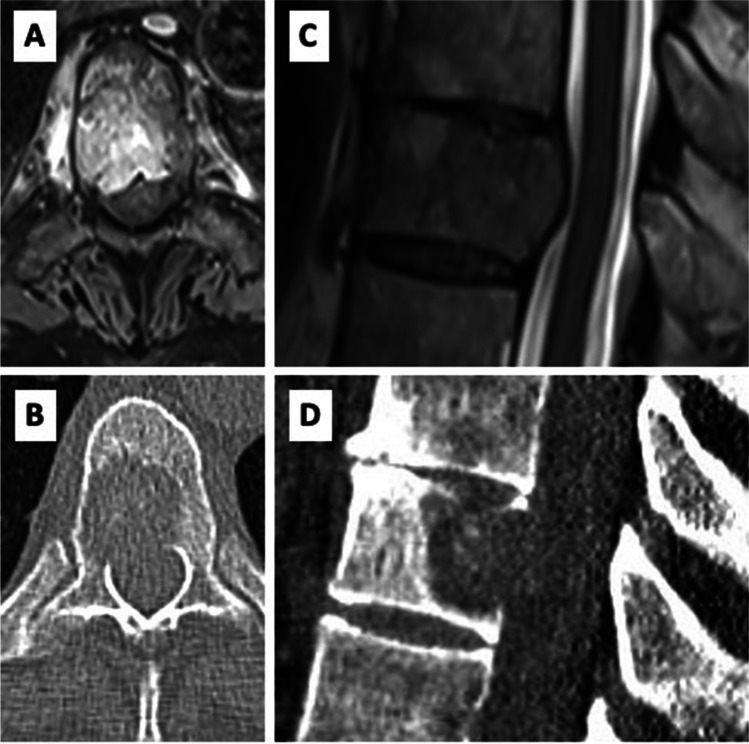
Baseline imaging **A**) axial MRI T1+Gd **B**) axial CT **C**) sagittal MRT T1-Gd **D**) sagittal CT

**Fig. 7:**
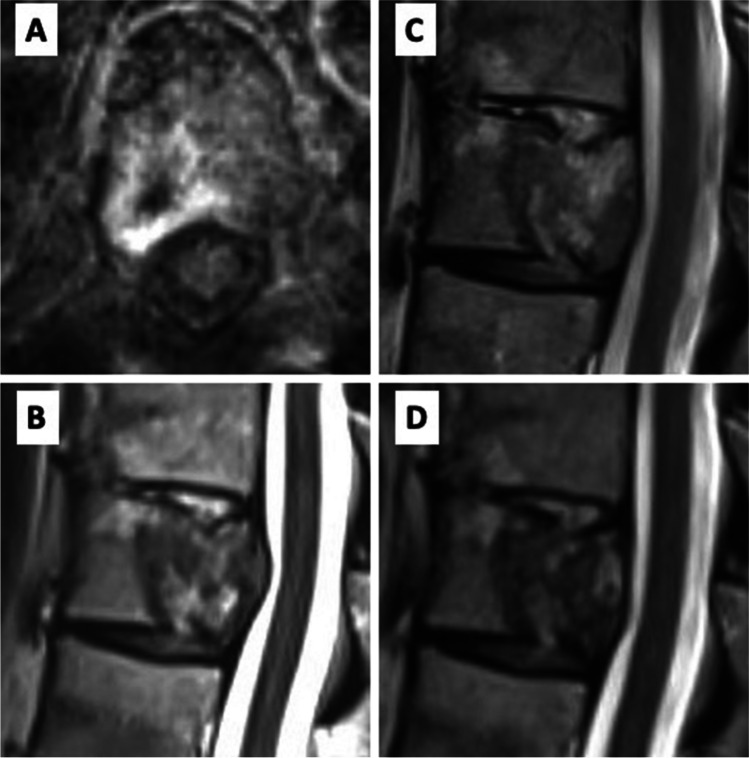
**A**) axial MRI T1+Gd 72h after surgery, **B**) sagittal MRI T2 after 3 months **C**) sagittal MRI T2 after 6 months **D**) sagittal MRI T2 after 10 months

Female 68-year-old patient with known metastatic (N1 M oss, pul) breast cancer. The patient suffered from several weeks long axial, non-radiating thoracic back pain (NRS 6/10), without neurological deficits. Imaging showed a new osteolytic metastasis at T10 (SINS 10), ESCC of 2c (Fig. 6). Surgery was performed via percutaneous ioCT navigated dorsal instrumentation of T9-11 using carbon implants, with a navigated transpedicular biopsy of the T10 vertebral metastasis and IORT was applied as described above. 8 Gy were dosed to a tissue depth of 1 cm from the tip of the applicator. Duration of surgery was 115 min., including 5 min. radiation time. The postoperative course was uneventful and the patient was discharged home 5 days after the intervention. The postoperative CT and MRI showed no signs of screw or rod displacement with adequate decompression of the spinal cord (Fig. 7A). After 3 weeks, the patient was pain-free and not taking any painkillers. Follow up at 3-, 6- and 10-months including imaging studies showed no signs of local recurrence and the patient remained pain-free and without any clinical signs instability (Fig. 7B, C, D respectively). ESCC remained 1.

## Discussion

We present a novel technique for spinal stabilization and IORT of unstable SM using one procedure only, describing the direct postoperative outcome and report AEs.

### Baseline parameters

Mean age of the study population was 71 years and therefore older than previous studies of IORT in spine patients [15, 21]. Mean age of patients affected by SM is 60 years [22]. This age difference might be due to our status as tertiary oncologic center where complex patients are treated. In our case series we performed IORT as an adjunct to spinal stabilization surgery in the same session. All patients were considered eligible for surgery by local anesthesiologic guidelines. As the criteria for a patient to be eligible for surgery are much stricter than the ones for RT, all patients who underwent surgery were in fact considered eligible for IORT. No patients with acute spinal cord compression or tumor mass infiltrating the pedicle were not treated with IORT.

### Surgical/IORT parameters

The integrative concept presented here aims to introduce a minimal invasive therapy option for multimetastatic patients with limited, unstable spinal metastases. The approach described in this manuscript has to be clearly distinguished from the concept of separation surgery, with its main goal of creating a safe zone between tumor and SC to enable RT and to limit radiation induced damage to the SC [6]. In painful spinal metastases, minimal invasive kyphoplasty has proven its efficacy as pain treatment and IORT in these patients during the same intervention using a similar technique [21, 23]. Whether surgical cement augmentation of the vertebrae supports the analgesic effect of IORT is unclear [24, 25]. In patients with (potentially) unstable spinal lesions often manifesting with mechanical pain, screw-rod stabilization surgery is warranted to treat pain but also prevent spinal deformity and neurological decline [26–28]. It has been shown that, after RT, up to one third of patients with multiple myeloma develop pathologic fractures during the following three years. This highlights the need for spinal stabilization in conditions with increasing life expectancy [29]. IORT is an option that enables comprehensive local treatment, mechanic stability, pain treatment and neurological preservation during a single session. This aims to create a less invasive setting by reducing potential radiation induced SSI during the postoperative course along with RT administration before a possible decline in general status and the postoperative recovery period. In cases with severe SC compression ESCC > 3 or involvement of the dorsal structures, IORT is not a therapeutic option and separation surgery might be considered. The use of IORT has prolonged surgery time by approximately 15 min including 5 min of actual radiation time. This increases if premature termination occurs due to bending of the needle applicator. Therefore, percutaneous surgery is considered in thoracic SM with a less lateral trajectory and in patients with less subcutaneous fat tissue as extensive soft tissue might give stress on the needle applicator. Especially in thoracic SM, it is important to reduce the tidal volume to a minimum during IORT as even ventilation might cause bending stress on the needle applicator.

### IORT

Intraoperative radiotherapy is a concept that has been applied in various ways and surgical indications for several years, e.g. breast cancer, colorectal cancer or brain tumors (metastases and glioma) [11, 12, 14, 30, 31]. In spinal surgery different techniques have been reported including brachytherapy, cone beam X-Ray radiotherapy and intratumorous X-Ray radiation. In the early 1990s, the concept of posterior decompression with IORT (PD-IORT) was developed. In spinal decompressions, open surgery was performed with dorsal decompression of the SC. Then a cone beam X-ray radiation was conducted after transferring the anesthetized patient to the RT facility whilst the surgical site remained open. The SC was covered with lead and the RT cone was inserted. This technique harbors the need for transfer of the anesthetized patient to the linac and was only applicable in open surgery. Previous studies also reported IORT on patients with serious motor deficits and epidural spinal cord compression [15, 32, 33]. On an individual basis, spinal instrumentation was performed and a SSI rate of 14% was reported.

More recently, a combination of kyphoplasty and IORT with the radiation emitting from within the center of the SM via a needle applicator was performed. In this technique, a 8-Gy radiation dose to the tumor border was calculated as maximum dose to the SC with much higher doses at the needle tip [21]. The feasibility of this technique has therefore been proven and a prospective trial is currently being performed [23]. Nevertheless, kyphoplasty might not be appropriate in case of spinal instability in which spinal stabilization is warranted.

In brachytherapy, capsuled radioactive agents are applied to the tissue, either via implanted tubes in an afterloading technique, or as permanent seeds and left in situ which expose neoplastic tissue to photon emission. This concept however brings practical issues of radiation safety to the OR which limits the handling and practicability during surgery. Brachytherapy in spinal surgery is rarely used nowadays but reports indicate its use in selected cases [34].

In present applications of low voltage IORT, radiation is applied to a resection cavity targeting the peritumoral infiltration zone. Depending on the size of the spherical applicator, longer radiation times are necessary to reach the cut of dose at the edge of the sphere that defines the border to the tissue. In the case of spine IORT the metastasis is left intact with a needle radiation applicator positioned at its center. Radiation doses are calculated to reach the critical dose limit of 8 Gy at the tumor border in order to respect the radiation threshold to the SC. With low energy X-Ray application, a steep radiation gradient is created between the needle tip and the “8-Gy line” resulting in local doses of several hundred gray in the center of the metastasis. This is in contrast to percutaneous RT where the tumor dose is limited by the proximity of the SC. One prior study on IORT performed radiation via a stamp like low-voltage X-Ray tube covering the SC with lead plates. This method did not make it to routine clinical use, possibly due to its complicated handling, poor radiotherapeutic options and the need for an open spinal approach. It is believed, that in the center which is exposed to high doses, RT causes radiation induced immunogenic cell death (ICD). Whether ICD can promote a tumor specific immune reaction, that might have systemic impact needs to be evaluated in future studies. We cannot draw any conclusions on the effect of this technique regarding the oncologic entity.

### Adverse events

Adverse events play a crucial role in oncologic surgery, as they can delay further treatment. In our cohort, nearly 50% of patients experienced at least one AE during the immediate postoperative course including serious AEs. These numbers need to be related to the overall reduced clinical state of patients suffering from metastasized diseases and surgery is regarded palliative. Even if the general state of patients decline after surgery, the RT for local control has already been administered. One major issue in oncological spinal surgery is SSI, which can result in fatal outcome. The rate of SSI after spinal instrumentation in SM is reported to lie between 4.5–20% [35–37]. Prior reports on IORT in open spinal surgery report a SSI rate of 14% [33]. On one hand, infections can be a life-threatening complication with repetitive surgical intervention. On the other hand, they can significantly delay further oncological therapy. Especially RT of the target area might lead to SSIs and wound healing should be completed before RT is applied on postoperative tissue [38].

### Future perspectives for intraoperative radiotherapy in surgery for spinal metastases

Local and minimally invasive therapy has a long history in neurosurgery. Stereotactic radioactive seed implantation has been previously performed for unresectable brain tumors. Stereotactic needle radiofrequency ablation is performed as part of so-called functional neurosurgery, for spinal denervation or targeted neuronal circuit modulation in the brain. More recently stereotactic laser interstitial thermal therapy has established itself as minimal invasive oncological therapy in cerebral neoplasms. Whether or not IORT in spinal metastases impacts local tumor control or even systemic tumor burden via a possible immune response needs to be established in a larger cohort with longer follow up. Our case series shows the feasibility of spine IORT with a reasonable complication rate. If this technique will lead to decreased hospital stays and economic benefits is the focus of currently ongoing studies. If so, especially in less dens populated countries or health care systems in second world areas, comprehensive local treatment with IORT might be an option to offer a more complete cancer therapy to patients without easy access to oncological treatment facilities.

### Limitations

We do have several limitations to report: this is a novel technique and the pitfalls we discovered during its establishment. As it’s the first time we have implemented this protocol, only limited number of heterogenous patients were treated. Treatment criteria were not clearly predefined and indication was extended to patients with ESCC > 2c. Follow-up was only 30 days as we focused on the immediate therapy related outcome and AE respecting the 30d period recommended by the CDC for spinal surgery. We however are planning on conducting a longer-term follow-up in these patients. As clinical practice, we started with selected cases. This data therefore represents the first description of our technique.

## Conclusion

We describe for the first time, spinal stabilization surgery with combined low energy IORT as treatment option in SM. This technique is feasible and safe but further studies on a larger population are needed to determine its role in the comprehensive oncological treatment of SM.

## Data Availability

Data is available upon request. Please address the corresponding author: philippemanuel.krauss@uk-augsburg.de.
